# Assessment of Cytogenotoxicity of Plastic Industrial Effluent Using *Allium cepa* Root Tip Cells

**DOI:** 10.1155/2023/5161017

**Published:** 2023-10-17

**Authors:** Jibril Sani Mohammed, Yahaya Mustapha, Madu Abdulkarim Him, Zandam Nuhu Danladi

**Affiliations:** ^1^Department of Biological Sciences, Federal University Dutse, Nigeria; ^2^Department of Plant Biology, Bayero University Kano, Nigeria; ^3^Department of Biological Sciences, Nigeria Defence Academy, Nigeria; ^4^Department of Science Laboratory Technology, Jigawa State Polytechnic, Dutse, Nigeria

## Abstract

The effects of plastic effluent in Kano Metropolis on cytotoxicity and genotoxicity were examined using a test on *Allium cepa* root cells. The physicochemical characteristics of industrial wastewater were assessed, and the results showed values that were higher than the required criteria; this implies that the effluent was not treated before to disposal. For 96 hours, a group of 40 onion bulbs was cultivated in various concentrations of plastic effluent: 15, 30, 45, and 60% (*v*/*v*). The control was made up of distilled water. Following 96 hours, the four treated root tips from each replication's bulbs were harvested and subjected to the acetoorcein squash technique for cytogenetic analysis. High concentrations of the industrial effluents had severe development retarding effects on the root tips. Root growth was inhibited with EC_50_ values of 48% after treatment with the effluents in comparison to control. When *Allium cepa* was exposed to different quantities of plastic effluent, the results of an analysis of variance (ANOVA) showed that the mean root length varied, and this variation was statistically significant (*p* < 0.05). With rising effluent concentrations, the mitotic index (M.I.) rapidly dropped. Chromosomal abnormalities were caused by the plastic effluent in the root cells of *Allium cepa*, especially sticky chromosome and binucleated cells being the most frequently seen at lower concentrations of 15%. It was discovered that the compounds found in plastic wastewater could injure live beings as well as harm the environment if not treated. Legal mechanisms must be used to push businesses and manufacturers to switch to environmentally friendly technologies.

## 1. Introduction

Due to the following benefits, onion (*Allium cepa* L.) is an excellent choice for cytogenotoxicity investigations. The onion has a constant chromosome number, a diversified chromosome architecture, a stable karyotype, and a clear and rapid response to genotoxic chemicals, and the occurrence of spontaneous chromosome damage is unusual. In addition, it has highly sensitive root development dynamics to contaminants, very clear mitotic phases, a stable karyotype, and a stable chromosome number. As a result, this test has established itself as a reliable method for identifying genotoxic chemicals in a variety of situations [1].

Other plants, most notably *Vicia faba* L. [2], maize [3], and *Drimia indica* Roxb. Jessop [4] have been used for cytogenotoxicity investigation. Aside from its long history of use in ecotoxicological tests, *Allium cepa* possesses a distinct advantage because of its huge chromosomes, which are simple to observe with a light microscope, as well as characteristics that may demonstrate an impact even at relatively small amounts of interaction of the evaluated substances on the genetic material itself. The findings from the *in vivo* experiment can be utilised to evaluate the genotoxicity of chemicals on plants as well as eukaryotes in general, including human beings. The utilisation of a plant, or a living being with a comparatively high degree of complexities, supplies data on potential DNA damage in a multicellular life, which cell cultures do not provide, even though findings may be significant because the genomes of cultured cells may be those of mammals, as in the case of a mouse bone marrow tissues [5, 6], or even turned cell lines from humans [7]. The *Allium test* is less expensive than using animals for testing [8–10] and has the potential to yield a significant amount of data using very basic methods of cultivation and without the ethical difficulties that surround animal experimentation and necessitate sophisticated breeding procedures. Additionally, Tedesco and Laughinghouse [8] demonstrated that when evaluating the effects of ecotoxicological drugs on animal cell lines and *Allium cepa*, the outcomes were equivalent and similar.

Many dangerous chemicals are present in the environment, and the majority of them are dumped into the soil, water, and air by industrial facilities. The world developed several chemical industries as a result of the ongoing use of chemicals. Toxins can infiltrate our environment through sources that are both natural and artificial [11]. They disturb a variety of biochemical reactions, which can have disastrous repercussions. They are also exceedingly difficult to eliminate from the environment once they have made their way into our biological systems. The genetic material has been altered and has undergone inheritable modifications as a result of numerous chemicals that have been evaluated as potentially mutagenic [12].

In this investigation, the cytogenotoxicity of industrial effluents discharged from the plastic industry in the Kano Metropolis will be assessed using the *Allium cepa* root cells. The findings of this work will help environmental regulatory organizations create *Allium cepa* root, a helpful tool in identifying mutagenic/clastogenic chemicals in industrial wastewater discharges and their presence and activity. This would therefore pave the way for investigations on the toxicity identification and assessment (TIA) of industrial effluents that were determined to be mutagenic. The main objectives of this study were to identify the physicochemical components of plastic manufacturing effluent, examine the impact of varying effluent concentrations on the root growth of *Allium cepa*, investigate the effects on various effluent concentrations on cytotoxic parameters associated with *Allium cepa* root cells, and determine the various types of chromosomal abnormalities caused by varying quantities of plastic effluent in *Allium cepa* roots.

## 2. Materials and Method

### 2.1. Study Area

Since the beginning of time, Kano State's capital has been located in Kano City. It is located 840 miles from the Sahara Desert's edge at latitude 12.0° N and longitude 8.30° E in West Africa's semiarid Sudan savannah region. The average elevation of Kano is roughly 472.45 meters above sea level. Kano's temperature frequently ranges between 33°C and 15.8°C [13].

### 2.2. Effluent Collection

At the site of environmental discharge, the plastic effluent from the MC Plastic Company in Kano's Sharada industrial zone was collected in 20-litre plastic containers. The effluent was deposited into a large drainage near to the facility, where it drained into substantial bodies of water.

### 2.3. Collection and Preparation of Plant Material

Common onion bulbs in good health and of equal size were purchased from the Albasa market on Katsina Road in Kano City, Nigeria. The infected bulbs were identified and eliminated. The plant medium (*Allium cepa*) was dried in the sun for two weeks before the fresh meristematic tissues were carefully exposed by shaving off the old roots that were found at the very bottom of onion bulbs. To keep the primordial cells from dying, the bulbs were immersed in newly prepared distilled water. To remove excess water, the bulbs were removed from the distilled water and laid on blotting paper [13].

### 2.4. Preparation of Effluents and Experimental Design

Following the preliminary trial, four concentrations of plastic effluent—15%, 30%, 45%, 60%, and 0% (*v*/*v*)—were created. A series of bulbs was then allowed to grow roots in distilled water. The onion bulbs with newly emerging roots were placed in 100 ml beakers filled with varying concentrations of each effluent after two days and left there for four days (or 96 hours). Daily changes were made to the test liquids [13]. There were a total of fifty (50) onion bulbs cultivated in a 100 ml glass beaker as part of the experiment, which had three replications and a complete randomized design (CRD).

### 2.5. Determination of Physicochemical Properties

Standard analytical protocol [14–16] for effluent discharge regulation was used to determine the standard physical and chemical properties of the effluent sample, such as temperature, pH, colour, dissolved oxygen (D.O.), biological oxygen demand (B.O.D.), total dissolved solids (T.D.S.), and heavy metals. The core laboratory of Bayero University Kano performed physicochemical analyses. However, atomic absorption spectrum (AAS) was used in the Centre for Dry Land Agriculture (CDA) to conduct heavy metal analysis. Before usage, the samples were stored in the refrigerator.

### 2.6. Cytogenetical Investigation

#### 2.6.1. Fixation

The bulbs were carefully rinsed with distilled water after treatment. Each bulb's root tips were removed and left in Carnoy's fluid (glacial acetic acid : ethanol: 1 : 3) for 24 hours.

### 2.7. Preparation of Squash

After hydrolyzing in 1N HCl for 1 minute at 60°C, the root tips were then placed in a magnifying glass contained aceto-orcein and 1N HCl (9 : 1) for chromosomal analysis. They were then steadily heated for 5-10 minutes before being covered and placed aside for 20-30 minutes. After that, a coverslip was placed on the glass slide with the tip of the root, which had been sliced with a sharp razor blade and immersed in a drop of 45% glacial acetic acid. A matchstick was used to tap down the root tip, which was then sealed with DPX. For several chromosomal abnormalities, the cells were examined under the microscope [11].

### 2.8. Determination of Root Growth/Inhibition

A commonly used approach for determining *Allium cepa* root length inhibition was utilised [17].

A calibrated ruler was used to measure the root lengths after the first, second, third, and fourth days of the experiment for both the control and treatment groups, and the mean values were computed. The effective concentration (EC_50_) was calculated on the graph at a point that indicated 50% growth after setting the mean root length of the control group to 100% and plotting the lengths of several treatment categories versus effluent concentrations. Using the formula indicated below, the percentages of the root length inhibition along with growth from all of the test effluents were calculated.

(1) $$\begin{array}{c} \text{Root length growth }\left(\%\right)=\frac{\text{root length of treatment group}}{\text{ root length of control}}\times 100,\\ \text{Root length inhibition }\left(\%\right)=\frac{\text{root length of control}-\text{root length of treatment }}{\text{root length of control}}\times 100. \end{array}$$

### 2.9. Evaluation of Cytotoxicity

The cytotoxic extent of the experimental substance was estimated using mitotic parameters (Mp) in the root cells of *Allium cepa*, such as mitotic index and mitotic inhibition. These were evaluated using the methods described by Bakare et al. [18]. Calculated values were expressed as percentages.

(2) $$\begin{array}{c} \text{Mitotic index}=\frac{\text{number of dividing cells}}{\text{ total number of cells obsreved}}\times 100,\\ \text{Mitotic inhibition}=\frac{\text{mitotic index in control group}-\text{mitotic index in test group}}{\text{mitotic index in control group}}\times 100,\\ \text{Frequency of aberration}=\frac{\text{total number of aberrant cells}}{\text{ total number cells count}}\times 100. \end{array}$$

### 2.10. Genotoxicity Determination

At various phases of the cell's cycle, including binucleated cells, vagrant, stickiness, bridge, laggard, and C-mitosis, photomicrographs of a few chosen cells exhibiting chromosomal aberrations were taken.

### 2.11. Data Analysis

The results were provided as three replicates of each sample's mean ± standard error (SE). The level of 5% was used to determine the statistical significance of differences between exposure treatments and the equivalent controls. Using the Excel application, analysis of variance (ANOVA) and post hoc least significant difference (LSD) were employed to see if there were any statistically significant differences between the measured parameters in Allium cepa roots exposed to the various effluent concentrations. Using Paleontological Statistics and Software (PAST), a plot of root length as a proportion of control versus sample concentrations was used to compute the EC_50_ and regression equation. Pearson's correlation analysis was used to assess for a meaningful link (positive or negative) between root length and effluent concentrations.

## 3. Results and Discussion

### 3.1. Physicochemical Characteristics

The physicochemical characteristics of plastic effluent are listed in Table 1, and the values were above the permissible limit for effluent discharge into the environment, although some were found to be within the limit. The biological oxygen demand and dissolved oxygen values were very low compared to allowed norms, which suggests that these industries generated a significant amount of organic pollutants, which are wastes with a substantial oxygen demand. The pH of plastic effluent was acidic. Heavy metals such as zinc, copper, iron chromium, and lead were detected in the effluent, and the levels were found to be above the permitted limit, demonstrating that the effluent was not treated before disposal. Metal poisoning is a serious environmental problem, especially in aquatic areas. Some metals are dangerous to humans if they enter the food chain because they may be poisonous or carcinogenic even at extremely low concentrations [19]. Heavy metals contained in plastic effluents such as Zn, Cr, Cu, and Pb may adhere to certain proteins in plants and aquatic organisms, restricting the functioning of membranes, metabolic processes in cells, and the movement of ions. Poisonous materials can sometimes prove fatal to aquatic species [20].

### 3.2. Morphology of Root Growth

**Figure 1:**
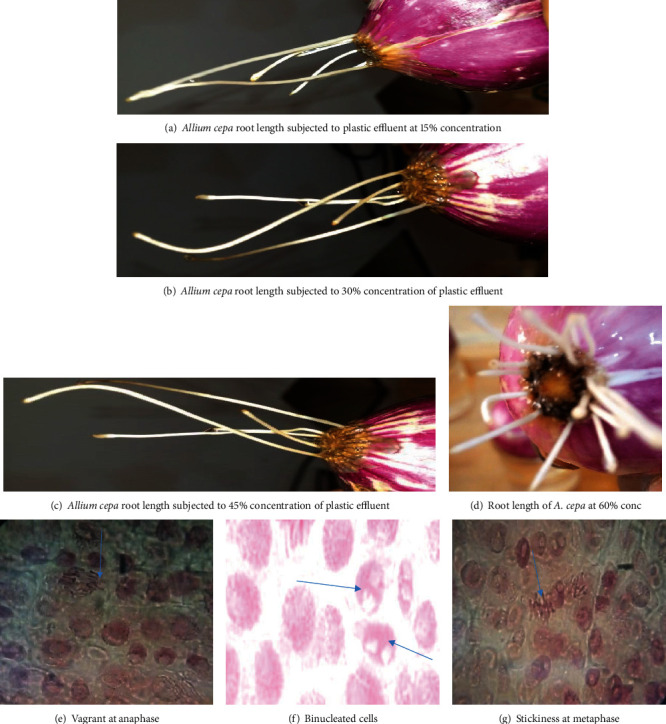
Root morphology and photomicrographs of cells showing chromosomal aberrations at different phases of the cell division.

**Figure 2:**
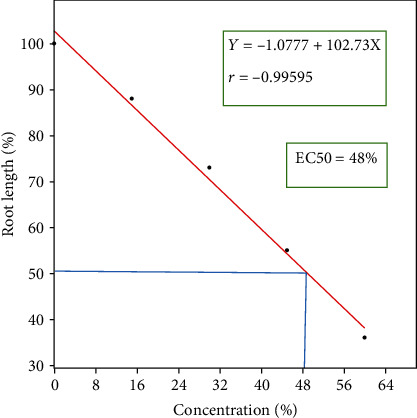
Roots of *Allium cepa* exposed to varying amounts of plastic effluent from the MC Plastic Company in Sharada, Kano, showed growth inhibition according to the Pearson correlation coefficient.

When exposed to varying amounts of plastic effluent, the root tips of Allium cepa displayed a number of distinct deformities, namely, puffy, puckered, and crotchet roots shown in Figure 1. The occurrence of root distortion in *Allium cepa* has signified an accurate indication of cytotoxicity [21]. The mean root lengths of the *Allium cepa* exposed to multiple amounts of the plastic effluent varied significantly, according to a statistical analysis using ANOVA (*p* < 0.05). It was generally found that the root growth inhibition study utilising Pearson's correlation shown in Figure 2 (*r* = −0.99595, *Y* = −1.0777 + 102.73*X*) was negatively associated with concentration, demonstrating that root development regression was highly concentration-dependent. Plastic effluent demonstrated cytotoxic effects on the roots of *Allium cepa* in accordance with the outcomes of the EC_50_ estimation as well as the root growth investigation. *Allium cepa* root cells exposed to different amounts of plastic effluent reported an EC_50_ value of 48 percent in Table 2 indicating that the substance was poisonous and hazardous. This might prevent cell division, which would result in phytotoxicity.

### 3.3. Cytotoxicity

With rising plastic effluent concentrations, it was discovered that the index of mitotic proliferation of *Allium cepa* root cells decreased (the mitotic index ranges from 32, 24, 20, 15, and 10 in 0%, 15%, 30%, 45%, and 60% of the plastic effluent as shown in Table 3). This outcome is consistent with the findings of Jibril et al. [13], who discovered that when the concentration of pharmaceutical effluent samples increased, the mitotic index, which reflects the percentage of diving cells, reduced. It was discovered that the control groups' mitotic indexes were higher than those of the treatment groups. In the control groups (those given distilled water treatment), there was no mitotic inhibition found. A useful statistic for quantifying cellular proliferation and estimating the proportion of cells that undergo mitosis is the mitotic index [22]. According to Haq et al. [23], the mitotic index (M.I.) is a useful biological monitoring technique for determining how different pollutants affect division of cells. When M.I. is inhibited, it may indicate cellular death since it gauges the percentage of cells that are in the mitotic stage of cell cycles [24]. A 50% fall in the index of mitotic activity relative to control is taken as the upper limit; a drop of less than 50% has sublethal impacts on the tested organism, while a decline of less than 22% has lethal consequences [25]. In this investigation, the plastic effluent sample reduced the index of mitotic activity, indicating both sublethal and fatal impacts in the cells of the onion root tip. At 60% concentrations, which might have a fatal effect, plastic effluent was extremely cytotoxic.

### 3.4. Genotoxicity

Table 4 provides a summary of the microscopic analysis of root cells of *Allium cepa* exposed to varying plastic effluent concentrations. According to chromosome analysis, the effluents created chromosomal abnormalities that were statistically significant (stickiness, bridge, laggard, vagrant, scattered, and binucleated cells) as in comparison to control. The chromosome of *Allium cepa* subjected to the control (distilled water) showed no abnormalities, and C-mitosis was not noticed. The binucleated cells and sticky chromosomes were found to be the most frequent aberrations at a concentration of 15% plastic effluent, respectively, with the bridge chromosome coming in third. Laggard, vagrant, and scattered chromosomes were those with the fewest abnormalities as shown in Figure 1. The results of the present investigation are congruent with previous research by Jibril et al. [13], who previously studied pharmaceutical industry effluents. The huge number of sticky chromosomes during the anaphase and metaphase stages demonstrated that industrial effluents contain hazardous compounds. Having a sticky surface on a chromosome is a sign that it has been poisoned, and this could cause cell death [26]. A disrupted nucleic acid metabolism may be the cause of stickiness [27]. During both the anaphase and metaphase stages of cell division, the genotoxic effect was primarily evident. The frequency of aberrations was concentration-dependent, meaning that it decreased as concentrations rose.

## 4. Conclusion and Recommendation

According to the current study, the plastic effluent has unacceptable concentrations of total dissolved solids (T.D.S.), conductivity, biological oxygen demand (B.O.D.), dissolved oxygen (D.O.), and pH, plus a high concentration of iron as well as substantial doses of various heavy metals such as chromium, copper, zinc, and lead. The effluents are harmful since these levels are over the permitted limit that environmental regulatory organizations propose as the upper limit. The research also showed that rising plastic effluent concentrations caused cytogenotoxicity, which in turn reduced the number of dividing cells, root development, index of mitotic activity, and total frequency of chromosomal abnormalities. Enforcing legislative regulations that require businesses to run ecofriendly operations is essential for promoting environmental sustainability.

## Figures and Tables

**Table 1:** Physicochemical characteristics of plastic industrial effluent collected from MC Plastic Company.

Parameters	Plastic effluent	FEPA^a^	USEPA^b^	WHO^c^
Colour	Cleared	NI	NI	NI
Odour	Not detected	NI	NI	NI
pH	6.2 ± 0.115	6.0-9.0	6.0-9.0	6.5-9.5
Temp	30.2 ± 0.577	<40	NI	NI
D.O.	3.4 ± 0.05	NI	NI	NI
T.D.S.	1964 ± 1.155	2000	500	<1200
B.O.D.	0.1 ± 0.0098	50	NI	NI
Conductivity	8590 ± 1.155	NI	NI	1200
Zinc	1.27 ± 0.0058	<1	0.12	NI
Iron	7.17 ± 0.58	2.0	0.009	3.0
Copper	1.39 ± 0.0058	<1	0.30	1.0
Lead	0.08 ± 0.0058	<1	0.003	0.01
Chromium	1.60 ± 0.058	<1	0.30	0.01

With the exception of the temperature, all measurements were in milligrams per litre; pH does not have a unit. ^a^Federal Environmental Protection Agency. ^b^United States Environmental Protection Agency. ^c^World Health Organization. NI: not indicated.

**Table 2:** Mean root length of *Allium cepa* exposed to different concentrations of plastic industrial effluent at 96 hrs.

Concentrations	R.L. (cm)	N.R.	% R.L.	Root G.I. (%)	EC_50_
Control	7.73 ± 0.15^a^	20 ± 0.58^a^	100	0	
15%	6.80 ± 0.15^b^	15.33 ± 0.33^b^	88	12	
30%	5.67 ± 0.32^c^	11.33 ± 0.33^c^	73	27	48%
45%	4.27 ± 0.32^d^	9.0 ± 0.58^d^	55	45	
60%	2.80 ± 0.15^e^	5.67 ± 0.33^e^	36	64	
LSD	0.73	1.41			

Significant differences between the means are indicated by different letters along the columns (*p* < 0.05). R.L.: root length; N.R.: number of roots; G.I.: growth inhibition; EC_50_: effective concentration at 50%.

**Table 3:** Mitotic characteristics of *Allium cepa* root cells exposed to varied amounts of plastic effluent after 96 hours.

Concentrations	T.N.C.	N.D.C.	M.I. (%)	M.Ih. (%)
Control	300	96[P_41_ M_25_ A_20_ T_10_] ± 2.85^a^	32	0
15%	300	72[P_30_ M_20_ A_14_ T_8_] ± 1.45^b^	24	25
30%	300	59[P_22_ M_16_ A_14_ T_7_] ± 1.53^c^	20	36
45%	300	45[P_15_ M_12_ A_13_ T_5_] ± 2.89^d^	15	53
60%	300	30[P_11_ M_8_ A_8_ T_3_] ± 2.89^e^	10	69
L.S.D.		7.62		

Significant differences between the means are indicated by the different letters along the columns. N.D.C.: number of dividing cells; T.N.C.: total number of cell count; L.S.D.: least significant difference; M.I.: mitotic index; M.Ih.: mitotic inhibition.

**Table 4:** Chromosomal abnormalities in *Allium cepa* root exposed to varied amounts of plastic effluent after 96 hours.

Concents	BIC	STK	BGE	LGD	VGT	SCT	C-MIT	F.O.A. (%)
Control	0.0	0.0	0.0	0.0	0.0	0.0	0.0	0.0
15%	3.0 ± 0.58	4.0 ± 0.58	2.0 ± 00	2.0 ± 0.58	1.7 ± 0.88	1.3 ± 0.88	0.0	4.7
30%	2.0 ± 0.58	3.0 ± 1.53	1.3 ± 0.33	1.0 ± 0.58	1.3 ± 0.33	1.7 ± 0.33	0.0	3.4
45%	1.7 ± 0.67	1.0 ± 0.58	1.0 ± 00	0.7 ± 0.67	0.7 ± 0.67	1.0 ± 00	0.0	2.0
60%	1.3 ± 0.33	1.3 ± 0.33	1.0 ± 0.58	0.7 ± 0.3	1.0 ± 0	1.0 ± 00	0.0	2.1

BIC: binucleated cells; STK: stickiness; BGE: bridge; LGD: laggard; VGT: vagrant; SCT: scattered; C-MIT: C-mitosis; F.O.A. (%): percentage frequency of aberrations; Concents: concentrations.

## Data Availability

The data is available upon request from the authors.
